# Steerable drops on heated concentric microgroove arrays

**DOI:** 10.1038/s41467-022-30837-z

**Published:** 2022-06-06

**Authors:** Cong Liu, Chenguang Lu, Zichao Yuan, Cunjing Lv, Yahua Liu

**Affiliations:** 1 Key Laboratory for Precision and Non-Traditional Machining Technology of Ministry of Education, Dalian University of Technology, Dalian, 116024 P. R. China; 2 Department of Engineering Mechanics and Center for Nano and Micro Mechanics, AML, Tsinghua University, Beijing, 100084 P. R. China

**Keywords:** Fluid dynamics, Wetting

## Abstract

Guided drop transport is of great importance in various water and thermal management technologies. Unidirectional drop transport on a hot surface has been widely developed, but a bidirectional reversal is still challenging. Here, we report a steerable transport of drop impinging on heated concentric microgroove arrays, on which the directionality of drop transport is dictated by the drop boiling modes. In the transition boiling state, the driving force originated from the Laplace pressure difference rendered by the microgrooves, which enables the drop rebounding towards the center of curvature. While in the film boiling state, a net force towards the opposite side is generated between the grooves and the penetrated liquid, that drives the drop far away from the center of curvature. Our experimental and theoretical results uncover that the lateral displacement is controlled by both the Weber number and off-center distance. These findings strengthen our fundamental understanding of drop impact dynamics at high temperatures and are essential for effective cooling of hot-spot cores and drop sieving.

## Abstract (web summary)

Guided drop transport at high temperature is of great importance in various water and thermal management technologies. Here, authors report a steerable drop transport dictated by the drop boiling states on heated concentric microgroove arrays.

## Introduction

Rectification of droplet transport on hot surfaces is both of fundamental interest and practical importance in spray cooling^1,2^, drag reduction^3–5^, and power generation^6,7^. In recent years, particular attention have been paid to the surface temperature above the so-called Leidenfrost point, where the drop levitates on its own vapor layer. Distinct from ambient conditions where the drop rectification is achieved by harnessing gradients of surface energy and often hindered by contact line pinning, in this Leidenfrost state, the underlying vapor layer of the drop allows the elimination of the contact line pinning and accordingly the associated interfacial friction. Therefore, various asymmetric structures such as macroscale or nanoscale ratchets, have been developed to realize unidirectional drop transport in the Leidenfrost state, resulting from the shear stress generated by the asymmetric vapor ejection^8–10^. However, the underlying vapor layer of the drop that allows negligible interfacial friction also causes a huge heat transfer resistance, which is adverse to effective thermal management.

Spray cooling, as an important cooling technique in many thermal management devices, can release the heat of devices by evaporating drops violently. The transient contact of the impinging drop on the hot surface is accompanied by transient phase change and intensive vibration. Thus, an impinging drop allows random guidance instead of full evaporation in situ, causing unsatisfactory cooling efficiency. Moreover, the temperature distribution tends to be asymmetrical on hot surfaces due to the random droplet transport, which in turn causes a thermal Marangoni effect^11,12^. Recently, researchers have proposed that directional drop rectification to the preferential region for enhanced heat transfer can be achieved on straight post arrays with a density gradient^1^ and regularly patterned posts with Janus-mushroom structures^13^. However, in all of these studies, the drop transport direction is not steerable for a fixed surface design, and the movement of drops are difficult to control quantitatively although the movement directions are explicit. Moreover, the unidirectional drop rectification relies on the elaborated surface structure design and scrupulous control of the specific location where the drop impacts. Therefore, controlled drop vectoring on hot surfaces remains a big challenge.

In this research, we design concentric microgroove arrays to achieve bidirectional drop transport by mediating the surface temperature, which goes beyond the traditional ways to realize unidirectional drop transport by chemical gradient^14,15^, ratchets^8,10^, and curvatures^16,17^. Impacting drop transports towards the center of curvature at temperatures below the Leidenfrost point, while towards the direction far away from the center of curvature at temperatures above the Leidenfrost point, i.e., the drop transport direction is dictated by the synergistic action of surface structure and boiling states. Simple scaling shows that the drop lateral transport distance depends on the Weber number and the off-center distance between the impact position and center of curvature. In addition, we demonstrate that this versatile and robust strategy could be applied in high-efficiency thermal transfer and drop sieving.

## Results

### Bidirectional drop transport at different boiling states

**Fig. 1:**
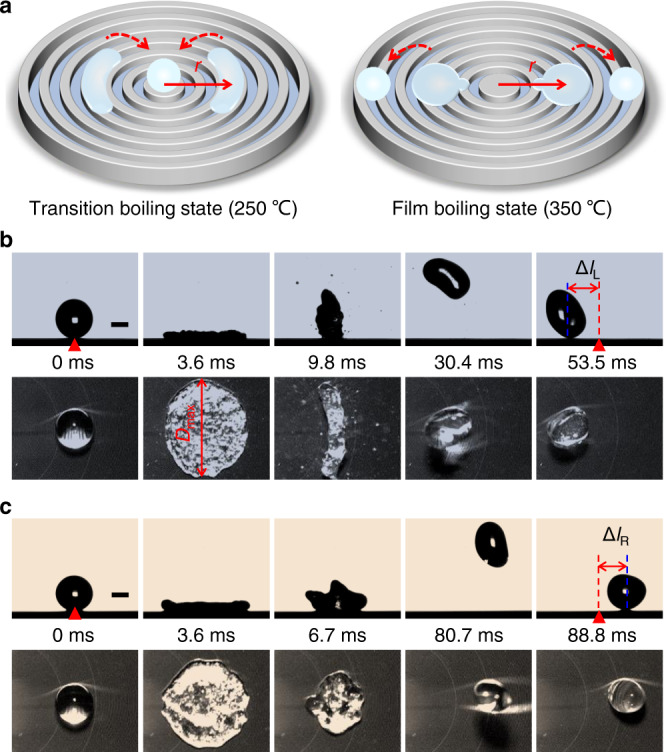
Drop dynamics in transition and film boiling states. **a** Schematic diagrams showing bidirectional motions of drops impacting on the heated concentric microgroove arrays at different temperatures. **b**, **c** Sequential images of the drop impact on the surfaces at *T* = 250 and 350 °C, respectively. Here, We = 16.9 and *r* = *d*/4. Δ*l*_L_ and Δ*l*_R_ denote the lateral displacements along the left and right directions, respectively. Scale bars showing in **b**, **c** are 1 mm.

The disc surface with a diameter *d* = 2 cm, as shown in the schematic diagram in Fig. 1a and Supplementary Fig. 1, consists of an array of concentric microgrooves with uniform ridge width *W* = 40 μm, groove width *S* = 40 μm, and height *H* = 20 μm, and the surface wettability at room temperature is discussed in Supplementary Note 1. We consider a water drop with radius *R* = 1.18 mm impinging on the surface with an off-center distance between the impact point and center of curvature *r* = *d*/4 under We = 16.9 at different temperatures. The Weber number is defined as We = *ρU*^2^*R*/*γ*, with *ρ* and *γ* being the mass density and surface tension of water, and *U* the impact velocity. At *T* = 200 °C, after the drop reaches its maximum spreading diameter, it boils violently until completely evaporates at 102.1 ms at its original location (Supplementary Fig. 3). In this case, the drop is in a contact boiling state that enables a high-efficiency heat transfer. When the temperature increases, i.e., *T* = 250 °C, a weak boiling is observed. Note that during recoiling, when the width of the drop along the radial direction reaches its minimum, the drop forms a bean shape (i.e., at *t* = 9.8 ms in Fig. 1b), and finally bounces towards the center of curvature, resulting in a lateral displacement Δ*l*_L_ = 1.84 mm between the impact point and the point where the drop falls back to the substrate (as shown in Fig. 1b and left column of Supplementary Movie 1). Surprisingly, the impinging drops converge into the center of curvature regardless of the releasing point (Supplementary Movie 2) at this temperature. When the temperature further increases, i.e., *T* = 350 °C, there is no obvious boiling, and the drop bounces back entirely, which is similar to that on superhydrophobic surfaces^18,19^. But note that, conversely, the drop bounces far away from the center of curvature with a lateral displacement Δ*l*_R_ = 1.76 mm, as shown in Fig. 1c and right column of Supplementary Movie 1.

**Fig. 2:**
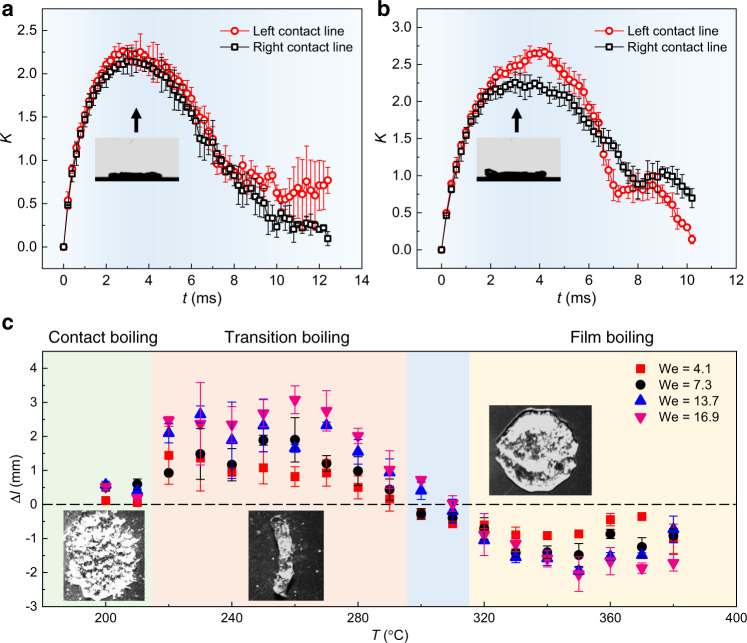
Displacement of the contact line and the drop. **a**, **b** Variation of contact line length ratio *K* of an impinging drop on concentric microgroove arrays (*r* = *d*/4) on the left (red) and right (black) sides under We = 16.9 at *T* = 250 and 350 °C, respectively. The insets in **a**, **b** represent the side view of maximum spreading. **c** Lateral displacements Δ*l* of bouncing drops as a function of temperature under various Weber numbers. A positive Δ*l*, i.e., Δ*l*_L_, indicates that drops rebound towards the center of curvature, while a negative one, i.e., Δ*l*_R_, towards the opposite direction. The insets show the recoiling of impacting drops shortly after they reach the maximum spreading at different boiling states. The error bars of the data in **a**–**c** denote the standard deviation of three measurements. Source data are provided as a Source Data file.

The directional drop transport is obvious when comparing the spreading and retracting processes during the impingement. Fig. 2a, b show the time evolution of normalized drop contact line length ratio *K* = *L*(*t*)/*R*, where *L*(*t*) represents the distance between the left/right contact point and the initial impact point over time. At *T* = 250 °C, as shown in Fig. 2a, the length ratios of the left and right contact lines are comparable with each other with the left one slightly larger, and finally, the whole drop bounces towards left. Note that, the right contact line recoils faster than the left one after *t* = 8 ms, due to the sheltering of the left side of the center rim by the front-most liquid. At *T* = 350 °C, however, the maximum length ratio of the left contact line is remarkably larger than the right one. After *t* = 4 ms, the left contact line recoils much faster than the right one (Fig. 2b), and finally, the drop bounces to the right.

We next plot the variation of the lateral displacement Δ*l* of the impinging drops as a function of the substrate temperature *T* for different We. As shown in Fig. 2c, when *T* is higher than the boiling point but less than 215 °C, an in situ contact boiling is observed, in which the drop is difficult to take off from the substrate until its complete evaporation. The slightly off-center distance results from measurement error due to the violent boiling of the drop. When the temperature increases, e.g., 215 °C ≤ *T* ≤ 295 °C, the impacting drop is in a transition boiling state, in which the drop always rebounds towards the center of curvature (Δ*l* > 0). However, when the temperature further increases, e.g., *T* > 315 °C, the drop is in a film boiling state, in which the drop always rebounds far away from the center of curvature (Δ*l* < 0). Note that, in the temperature range 295 °C ≤ *T* ≤ 315 °C, there is a transition during which a hybrid boiling exists, and the drop rebound direction is random with a relatively small lateral displacement. The drop would rebound to the left when the transition boiling dominates under a high Weber number, e.g., data points We = 13.7 and 16.9 at *T* = 300 °C, while to the right when the film boiling prevails under a low Weber number, e.g., data points We = 4.1 and 7.3 at *T* = 300 °C. This is because the Leidenfrost temperature increases with the Weber number and it is not a fixed value^20^.

**Fig. 3:**
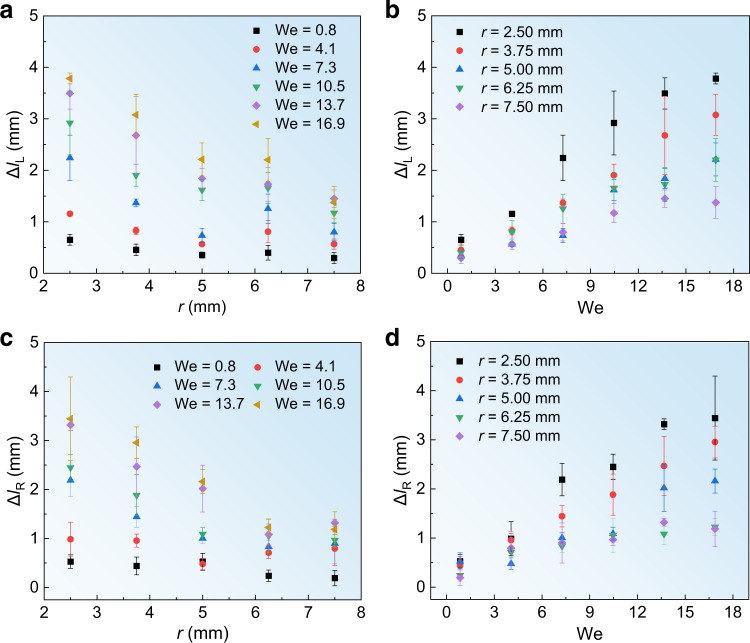
Lateral displacement Δ*l* as functions of *r* and We at different temperatures. **a**, **b** Variation of Δ*l*_L_ at *T* = 250 °C as a function of *r* under various We and as a function of We for various *r*, respectively. **c**, **d** Variation of Δ*l*_R_ at *T* = 350 °C as a function of *r* under various We and as a function of We for various *r*, respectively. The error bars of the data in **a**–**d** denote the standard deviation of three measurements. Source data are provided as a Source Data file.

Further, we investigate the influence of off-center distance *r* and Weber number We on the lateral displacement Δ*l* at different temperatures. As shown in Fig. 3a, b, in the transition boiling state at *T* = 250 °C, it is obvious that Δ*l*_L_ decreases with *r* for a fixed We, but increase with We for a specific *r*. Similar tendencies are observed in the boiling state at *T* = 350 °C, as shown in Fig. 3c, d. These facts suggest that, despite remarkably different temperatures of the substrates and rebounding directions of the drops, Δ*l*_L_ and Δ*l*_R_ are closely related to *r* and We.

### Mechanism of drop transport at transition boiling state

**Fig. 4:**
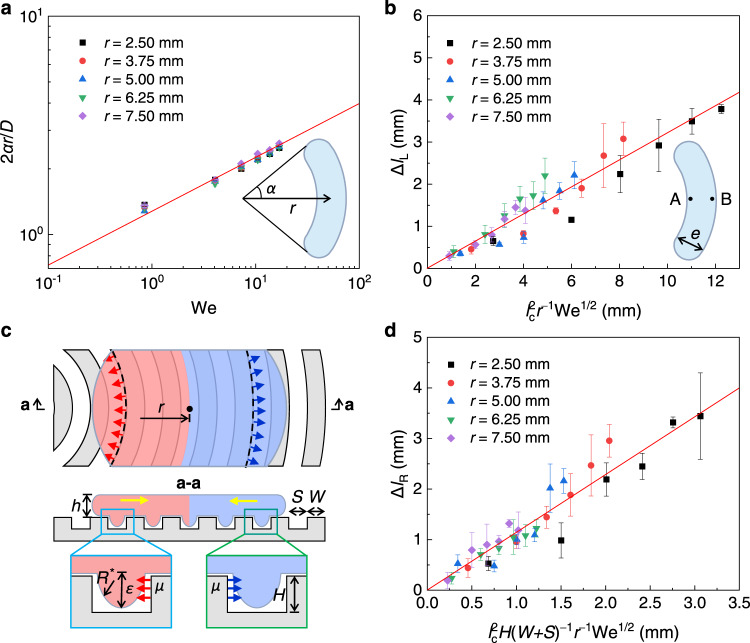
Mechanism for the lateral transport of drops in the transition boiling (a and b) and film boiling (c and d). **a** Relationships between 2*αr*/*D* and We. **b** The lateral displacement Δ*l*_L_ as a function of We and the initial off-center distance *r*. Here, *R* = 1.18 mm, and the red solid lines in **a**, **b** are respectively the best fits of Eqs. 1 and 3 to the experimental data. **c** The top panel sketching the morphology of the drop during recoiling from the top view. The red and blue arrows represent the forces that the grooves exert on the liquid. The bottom panel sketching the wetting state of the sections a-a. The left and right parts of the drop are marked by the light red and light blue shadows, respectively. **d** The lateral displacement Δ*l*_R_ as a function of We and *r*. The solid line is the best fit of Eq. 5. The error bars of the data in **a**, **b**, **d** denote the standard deviation of three measurements. Source data are provided as a Source Data file.

We now explore the underlying mechanisms accounting for the directional rebounding in the transition boiling at *T* = 250 °C. To address the question, we first consider the drop deformation in the spreading and receding processes, which plays an important role in the interaction between the drop and the substrate. As shown in Fig. 1b, violent boiling occurs when the drop contacts the substrate, which may render a partial wetting of the contact region. In addition, due to a smaller contact angle hysteresis along the circumferential direction compared with the radial direction of the substrate, the drop spreads larger along the grooves and evolves into an elliptical shape (i.e., 3.6 ms) rather than a perfect circle at its maximum extension. In this case, more liquid flows to the two ends along the circumferential direction, leaving a relatively thinner layer on the left and right sides. Since the recoiling drop dewets at the Taylor–Culick velocity which is inversely proportional to one-half of the thickness of the liquid layer^21,22^, the left and right sides recoil faster than the upper and lower ends, and finally, a bean-shaped drop is formed at the end of the retraction, i.e., at 9.8 ms (Supplementary Discussion 1). This behavior is remarkably different from that in previous research, in which an impacting drop recoils in a circular shape^23^ or evaporates immediately^20^. Note that the arc length of the bean-shaped drop at *t* = 9.8 ms is close to its maximum spreading width *D*_max_ at *t* = 3.6 ms (Fig. 1b), and therefore we have 2*rα* ≈ *D*_max_, where *α* is the corresponding central half-angle, as shown in the inset of Fig. 4a. Following the scaling^24^ of *D*_max_/*D* ~ We^1/4^, we obtain

1 $$\frac{2r\alpha }{D} \sim {{{\mathrm{We}}}}^{1/4},$$

where *D* = 2 *R* is the initial drop diameter. Indeed, this is observed for different values of *r* and We, where the experimental data is in good agreement with Eq. 1 with a prefactor 1.25 based on the best fit (Fig. 4a).

The driving force that propels the drop to the curvature center of the substrate could be deduced based on the asymmetric profiles of the drop. From the top view in the left column of Supplementary Movie 1, the asymmetry of the left and right profiles of the drop is particularly obvious in the recoiling state, resulting in the different curvatures on the left and right sides of the torus. Consequently, a Laplace pressure difference *δP* is rendered, which not only accounts for the asymmetric deformation of the drop but also the lateral motion of the entire drop. An exact deduction of *δP* in the whole recoiling stage is challenging due to the complex and changeable morphology of the drop. However, *δP* could be perceived based on the bean-shaped drop at 9.8 ms in Fig. 1b. In other words, *δP* in the recoiling stage must have a strength that is able to force the circular-shape drop into a bean-shaped one, and *δP* exerted on the bean-shaped drop could be considered as the characteristic value of which in the recoiling stage. In this regard, we make analyses based on the bean-shaped drop where there are two principal curvature radii, i.e., azimuthal radii *r*_1_ = *r* − *e*/2 and *r*_2_ = *r* + *e*/2 closed to the left (point A) and right (point B) sides of the liquid-vapor menisci (Supplementary Fig. 5), respectively, while meridional radii *e*/2 for both of the left and right sides (points A and B), where *e* is denoted as the width of the bean-shaped drop (inset of Fig. 4b). Based on the Young–Laplace equation^25^, a pressure difference between points A and B is obtained, i.e., *δP* = *P*_B_ – *P*_A_ ≈ *γ*(1/*r*_1_ + 1/*r*_2_) > 0, which renders an inner pressure difference that propels the drop to move to the left side. Since *e* ≪ *r*, *δP* ≈ *γ*(1/*r*_1_ + 1/*r*_2_) ≈ 2*γ*/*r* ~ *γ*/*r* is expected (Supplementary Discussion 2). Considering *δP* works along the horizontal direction, the force exerting on the drop along the horizontal direction is *F*_L_ = *δP*·*A*, where *A* ~ *D*_max_^2^ characterizes the contact area between the solid and liquid interfaces in the recoiling stage rather than the specific moment of the triggered bean shape. Finally, we obtain

2 $${F}_{{{\mathrm{L}}}} \sim \delta P\cdot {D}_{\max }^{2} \sim \frac{\gamma }{r}\cdot {R}^{2}{{{\mathrm{We}}}}^{1/2}.$$

Thus, the acceleration along the lateral direction that propels the drop to the center of the curvature is characterized as *a*_L_ = *F*_L_/*m*, with *m* = 4π*ρR*^3^/3 being the mass of the drop. Accordingly, the corresponding lateral speed could be estimated as *V*_L_ = *a*_L_*τ*_0_, with *τ*_0_ ~ (*ρR*^3^/*γ*)^1/2^ being the classic solid-liquid contact time^23^ during the drop impingement. Considering a free falling of the drop after detaching from the substrate with an upward speed ${U}_{\perp }$, the horizontal flying time span is $\varDelta t \sim {U}_{\perp }/g$, with *g* being the acceleration of gravity. Here, ${U}_{\perp }$ is the characteristic rebounding velocity^26^ and could be estimated as ${U}_{\perp }={[\gamma /(\rho R)]}^{1/2}$. Finally, we have $\varDelta {l}_{{{\mathrm{L}}}} \sim {V}_{{{\mathrm{L}}}}\varDelta t \sim ({F}_{{{\mathrm{L}}}}{\tau }_{0}/m)({U}_{\perp }/g)$ and a combination of Eq. 2 leads to

3 $$\varDelta {l}_{{{{{{\rm{L}}}}}}} \sim {l}_{{{\mathrm{c}}}}^{2}\frac{{{{\mathrm{We}}}}^{1/2}}{r}.$$

In Eq. 3, *l*_c_ = [*γ*/(*ρg*)]^1/2^ is defined as the capillary length, which is about 2.73 mm for water in the ambient laboratory environment. As shown in Fig. 4b, the lateral distance Δ*l*_L_ estimated by Eq. 3 is very well consistent with the experimental data, with a prefactor 0.32 based on the best fit.

### Mechanism of drop transport at film boiling state

We now explore the underlying mechanism accounting for the lateral displacement of the drop in the film boiling state. Different from the transition boiling state, the temperature of the film boiling state is higher, and a thin layer of air film is generated underneath the drop that avoids direct contact between the liquid and solid substrate. Moreover, the existence of the air film significantly reduces the adhesion between the drop and the grooves, which endows a circular shape of the drop during the whole impingement (Fig. 1c). Here, we hypothesize that the force propelling the drop to move laterally would be a result of the interaction between the liquid and the substrate. To address this point, we first check the morphology of the liquid in the contact region. Upon impact, a balance reaches between the dynamic pressure *ρU*^2^/2 and the capillary pressure *γ*/R^*^, resulting from the dynamics of the drop and the liquid-vapor meniscus generated in the grooves, respectively. Here, R^*^ ~ *S*^2^/*ε* represents the curvature radius of the liquid-vapor meniscus in the grooves^27^, with *ε* being the penetration depth. The above analysis leads to an estimate of the penetration depth, i.e., *ε* ~ *S*^2^We/*R* (see details in Supplementary Discussion 3). The parameters used in our experiments (i.e., *S* = 40 μm, *R* ~ 1 mm, and We ~ 10) give rise to *ε* ~ 10 μm, whose order is comparable to the depth of the groove (*ε* ~ *H*) and suggests a penetration of the liquid into the grooves, and therefore, the recoiling liquid will exert forces on the inner wall of the grooves during the drop retraction process. In turn, the inner wall of the grooves will exert equivalent forces on the liquid in the opposite direction, as represented by the red and blue arrows in Fig. 4c. To give a quantitative analysis, we use *μ* to denote the aforementioned force per length (N/m) in a single groove, i.e., *μ* ~ *ρU*_//_^2^*H*, with *U*_//_ ~ [*γ*/(*ρh*)]^1/2^ and *h* = *R*We^−1/2^ being the characteristic lateral velocity and thickness of the recoiling liquid, respectively^28^. After integration over the whole contact region, the resultant acting force could be obtained (see details in Supplementary Discussion 4). For simplicity, we consider the drop in two parts, i.e., the left and right parts which are marked by the red and blue colors, respectively. On the left side of the drop (red shadow in Fig. 4c), the acting force on the drop from the grooves could be estimated as *F*_left_ ≈ 2*μ*(*R*^2^ − *R*^3^/*r*)/(*W* + *S*). Similarly, the force on the right (light blue shadow in Fig. 4c) is *F*_right_ ≈ 2*μ*(*R*^2^ + *R*^3^/*r*)/(*W* + *S*). Moreover, the net force *F*_R_ = *F*_right_ − *F*_left_ exerted on the drop is obtained as

4 $${F}_{{{\mathrm{R}}}} \sim \frac{{R}^{2}H\gamma }{W+S}\cdot \frac{{{{\mathrm{We}}}}^{1/2}}{r},$$

which points to the right and enables the drop to bounce far away from the center of the curvature. Considering $\varDelta {l}_{{{\mathrm{R}}}} \sim {V}_{{{\mathrm{R}}}}\varDelta t \sim ({F}_{{{\mathrm{R}}}}{\tau }_{0}/m)({U}_{\perp }/g)$ as analyzed before, we finally obtain

5 $${\varDelta {l}}_{{{\mathrm{R}}}} \sim \frac{H{l}_{{{\mathrm{c}}}}^{2}}{W+S}\cdot \frac{{{\mathrm{We}}}^{1/2}}{r}.$$

Equation (5) suggests that the lateral displacement Δ*l*_R_ of the drop impacting the concentric microgroove arrays at the film boiling state is also controlled by both the Weber number and the initial off-center distance, which is well confirmed by the data in Fig. 4d. The prefactor of Eq. 5 obtained from the best fit to the experimental data is 1.15. In addition, the obtained forces *F*_left_ and *F*_right_ in the above give us fundamental insights to understand the drop spreading and recoiling dynamics in Fig. 2b. The above result *F*_left_ < *F*_right_ indicates a smaller resistance exerted on the liquid, that the drop spreads faster and the contact length is larger to the left than that to the right. On the contrary, the left contact line moves faster than the right one during the recoiling stage.

To further validate the universal applicability of the steerable drop transport method by employing concentric microgroove arrays, as well as the proposed models, systematic experiments were conducted to elaborate on the effect of structural parameters on the drop lateral transport distance (see details in Supplementary Discussion 6). Specifically, substrates with different ridge width *W* (20 and 40 μm), groove width *S* (10, 40, and 80 μm), and ridge height *H* (10, 20, 50 μm) were fabricated, and labeled as W20S10H20 (i.e., *W* = 20 μm, *S* = 10 μm, *H* = 20 μm), W20S40H20, W20S80H20, W40S10H20, W40S40H20, W40S80H20, W40S40H10, and W40S40H50, respectively (Supplementary Fig. 9). It is obvious that the proposed approach is well validated and the lateral displacement of impacting drops on different substrates follows the same scaling laws, e.g., Δ*l*_L_ ~ *l*_c_^2^*r*^−1^We^1/2^ in the transition boiling state and Δ*l*_R_ ~ *l*_c_^2^*H*(*W* + *S*)^−1^*r*^−1^We^1/2^ in the film boiling, respectively, as shown in Supplementary Fig. 10.

### Cooling abilities and drop sieving

**Fig. 5:**
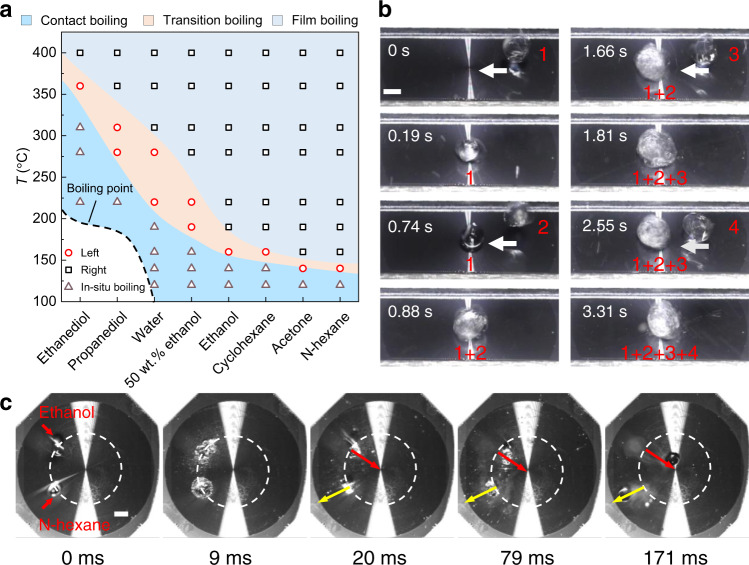
Bidirectional transport of various liquids and drop sieving. **a** Phase diagram for different transport directions of various liquids at different temperatures. “Left” represents the drop rebounding towards the center of curvature, while “Right” represents the drop rebounding far away from the center of curvature. **b** Sequential images showing the convergence of successive water drops on the center of curvature at *T* = 250 °C and We = 10.5. **c** Comparison between opposite rebounding directions of ethanol and *n*-hexane drops showing the ability of drop sieving at *T* = 160 °C, We = 10.5, and *r* = *d*/4. The ethanol drop bounces towards the center of curvature, while the *n*-hexane drop bounces far away from the center of curvature. Scale bars in **b**, **c** are 2 mm.

The ability to deposit drops to obtain desired transport directions in a highly repeatable manner is desired for thermal dissipation with high efficiency, and can also be utilized to perform practical tasks such as drop sieving for fluidic-based applications^29,30^. The advantages of our work will largely benefit these concepts, which will be illustrated as follows. It is well-known that the boiling state of the liquid as a function of the substrate temperature is related to the liquid property^31,32^, and Fig. 5a shows a phase diagram describing the bidirectional transport of various liquids (Table S1) at different temperatures. First, for a specific value of the substrate temperature, we could use a liquid to guarantee that the impinging drop is always in the transition boiling state. As shown in Fig. 5b, when successive water drops are deposited, they can be converged in the center of curvature (Supplementary Movie 3), which is promising to achieve a high-efficient heat transfer at the center of the substrate, especially in cooling hot spots. Second, as shown in Supplementary Fig. 12a and Supplementary Movie 4, by leveraging the opposite rebounding direction of two kinds of droplets with different liquid properties (for example, ethanol and *n*-hexane) at a certain temperature, e.g., *T* = 160 °C, the synchronously deposited drops rebound to opposite directions to realize drop sieving (Fig. 5c and Supplementary Movie 5). These intriguing transport behaviors on our surfaces are general, and more results are given in Supplementary Fig. 12b–d and Supplementary Movies 6–8.

## Discussion

In summary, we have reported a strategy to steer drops on hot concentric microgrooved substrates. In the transition boiling, the impinging drop forms a bean shape during the retraction process, which generates a Laplace pressure difference, driving the drop towards the center of curvature. In the film boiling state, however, the impacting drop rebounds towards the opposite direction, i.e., far away from the center of curvature, owning to the interaction force between the penetrated liquid and the grooves. This work breaks through our understanding that drops on hot textured surfaces could only be transported in one direction, such as the Leidenfrost drops. Recently, Jiang et al. report a remarkable phenomenon that the Leidenfrost effect could be inhibited up to a record-high temperature^33^, e.g., 1100 °C, which is rationalized by constructing the termed “structured thermal armor” to decouple the wetting phenomena from the vapor dynamics. Here, we propose an alternative approach that emphasizes the richness of thermal management. This steerable and highly repeatable manner to realize directional transportation of drops, as well as detailed fundamental understandings of the physics that we have revealed, provides a paradigm to manipulate drop transportation on hot surfaces available in various applications, including spray cooling and drop sieving, among many others.

Still, some open questions remain. For example, the contact state due to boiling modes between the liquid and the substrate during the impingement plays a crucial role in dictating the drop rebound dynamics, however, direct observation of the contact state on our structured surface challenges the current technology^20^, but which is essential for a scientific breakthrough. In the present experiment, hydrophilic substrates are employed. Impingement on substrates with different wettabilities under even higher temperatures and Weber number remain to be further explored, which could be of considerable interest for practical applications. Moreover, the vertical component of the pressure resulting from the thin vapor film may affect the overall drop bouncing dynamics, and further investigation is still needed. Therefore, a thorough clarification of these influences accounting for the steerable drop transport would deserve a dedicated study in the future.

## Methods

### Fabrication of concentric microgroove arrays

The concentric microgroove arrays were fabricated using the standard photolithography technique on a silicon wafer. Specifically, a SiO_2_ layer with a thickness of 2 μm was first deposited on the silicon wafer with a thickness of 500 μm at high temperature. The photoresist was uniformly coated on the SiO_2_ layer. Then the uncovered SiO_2_ was etched by Reactive Ion Etching (RIE). Deep RIE was used to further etch the silicon substrate to form the micropattern. A series of substrates were fabricated with different ridge width *W* (20 and 40 μm), groove width *S* (10, 40, and 80 μm), and ridge height *H* (10, 20, 50 μm), and are labeled as W20S10H20 (i.e., *W* = 20 μm, *S* = 10 μm, *H* = 20 μm), W20S40H20, W20S80H20, W40S10H20, W40S40H20, W40S80H20, W40S40H10, and W40S40H50, respectively, as shown in Supplementary Figs. 1, 9.

### Drop impact experiments on hot substrates

The sample was heated on a hot plate, in which a thermocouple was used to monitor the surface temperature. Deionized water droplets of radius *R* = 1.18 mm were released from a syringe pump equipped with a fine needle at a flow rate of 2 μL/s. The height between the droplets and the substrates was adjusted to change the impact velocity *U* from 0.23 to 1.02 m/s, corresponding to the Weber number (We) of 0.8 < We < 16.9, where We = *ρU*^2^*R*/*γ* is defined as the Weber number with the liquid density *ρ* = 998 kg/m^3^ and the liquid surface tension *γ* = 72 mN/m. The drop dynamic behavior is filmed simultaneously from the side and top views using two synchronous high-speed cameras (Photron SA5) at a frame rate of 10,000 fps.

### Statistics and reproducibility

No statistical method was used to predetermine sample size. No data were excluded from the analyses. The experiments were not randomized. The Investigators were not blinded to allocation during experiments and outcome assessment.

## Supplementary information

Supplementary Information

Description of Additional Supplementary Files

Supplementary Movie 1

Supplementary Movie 2

Supplementary Movie 3

Supplementary Movie 4

Supplementary Movie 5

Supplementary Movie 6

Supplementary Movie 7

Supplementary Movie 8

## Data Availability

The data that support the findings of this study are available from the corresponding authors upon request. Source data are provided with this paper.

## Source data

Source Data

## References

[1] Li J (2016). Directional transport of high-temperature Janus droplets mediated by structural topography. Nat. Phys..

[2] Wendelstorf J, Spitzer KH, Wendelstorf R (2008). Spray water cooling heat transfer at high temperatures and liquid mass fluxes. Int. J. Heat. Mass. Transf..

[3] Vakarelski IU, Marston JO, Chan DYC, Thoroddsen ST (2011). Drag reduction by Leidenfrost vapor layers. Phys. Rev. Lett..

[4] Quéré D (2013). Leidenfrost dynamics. Annu. Rev. Fluid Mech..

[5] Vakarelski IU, Patankar NA, Marston JO, Chan DYC, Thoroddsen ST (2012). Stabilization of Leidenfrost vapour layer by textured superhydrophobic surfaces. Nature.

[6] Wells GG, Ledesma-Aguilar R, McHale G, Sefiane K (2015). A sublimation heat engine. Nat. Commun..

[7] Agrawal P (2019). Leidenfrost heat engine: Sustained rotation of levitating rotors on turbine-inspired substrates. Appl. Energ..

[8] Linke H (2006). Self-propelled Leidenfrost droplets. Phys. Rev. Lett..

[9] Dupeux G (2011). Viscous mechanism for Leidenfrost propulsion on a ratchet. Europhys. Lett..

[10] Lagubeau G, Le Merrer M, Clanet C, Quéré D (2011). Leidenfrost on a ratchet. Nat. Phys..

[11] Thimbleby H (1989). The Leidenfrost phenomenon. Phys. Educ..

[12] de Ruiter J, Soto D, Varanasi KK (2018). Self-peeling of impacting droplets. Nat. Phys..

[13] Liu M (2020). Inhibiting random droplet motion on hot surfaces by engineering symmetry-breaking Janus-mushroom structure. Adv. Mater..

[14] Chaudhury MK, Whitesides GM (1992). How to make water run uphill. Science.

[15] Ichimura K, Oh SK, Nakagawa M (2000). Light-driven motion of liquids on a photoresponsive surface. Science.

[16] Lv C (2014). Substrate curvature gradient drives rapid droplet motion. Phys. Rev. Lett..

[17] Ju J (2012). A multi-structural and multi-functional integrated fog collection system in cactus. Nat. Commun..

[18] Liu Y (2014). Pancake bouncing on superhydrophobic surfaces. Nat. Phys..

[19] Zhan H (2021). Horizontal motion of a superhydrophobic substrate affects the drop bouncing dynamics. Phys. Rev. Lett..

[20] Tran T, Staat HJJ, Prosperetti A, Sun C, Lohse D (2012). Drop impact on superheated surfaces. Phys. Rev. Lett..

[21] Taylor G (1959). The dynamics of thin sheets of fluid. III. Disintegration of fluid sheets. Proc. R. Soc. Lond. A.

[22] Culick FEC (1960). Comments on a ruptured soap film. J. Appl. Phys..

[23] Richard D, Clanet C, Quéré D (2002). Contact time of a bouncing drop. Nature.

[24] Clanet C, Beguin C, Richard D, Quéré D (2004). Maximal deformation of an impacting drop. J. Fluid Mech..

[25] Quéré D (2008). Wetting and roughness. Ann. Rev. Mater. Res..

[26] Reyssat M, Pardo F, Quéré D (2009). Drops onto gradients of texture. Europhys. Lett..

[27] Soto D, Lagubeau G, Clanet C, Quéré D (2016). Surfing on a herringbone. Phys. Rev. Fluids.

[28] Mouterde T (2019). Two recipes for repelling hot water. Nat. Commun..

[29] Feng S (2021). Three-dimensional capillary ratchet-induced liquid directional steering. Science.

[30] Mazaltarim AJ, Bowen JJ, Taylor JM, Morin SA (2021). Dynamic manipulation of droplets using mechanically tunable microtextured chemical gradients. Nat. Commun..

[31] Gottfried BS, Lee CJ, Bell KJ (1966). The leidenfrost phenomenon: film boiling of liquid droplets on a flat plate. Int. J. Heat. Mass. Transf..

[32] Biance AL, Clanet C, Quéré D (2003). Leidenfrost drops. Phys. Fluids.

[33] Jiang M (2022). Inhibiting the Leidenfrost effect above 1,000 °C for sustained thermal cooling Leidenfrost drops. Nature.

