# Supplementary material for: Steerable drops on heated concentric microgroove arrays
Source: Nat Commun. 2022 Jun 6;13:3141. doi: 10.1038/s41467-022-30837-z (PMC9170727; doi:10.1038/s41467-022-30837-z)
Supplement: Supplementary file 1 — Supplementary Information [file 41467_2022_30837_MOESM1_ESM.pdf]

Supplementary Information for

Steerable drops on heated concentric microgroove arrays

Cong Liu[1], Chenguang Lu[1], Zichao Yuan[1], Cunjing Lv[2]*, Yahua Liu[1]*

[1]Key Laboratory for Precision and Non-Traditional Machining Technology of Ministry of Education, Dalian University of Technology, Dalian 116024, P. R. China

[2]Department of Engineering Mechanics and Center for Nano and Micro Mechanics, AML, Tsinghua University, Beijing 100084, P. R. China

*Corresponding author: yahualiu@dlut.edu.cn (Y.L.) and cunjinglv@tsinghua.edu.cn (C.L.)

This PDF file includes:

Supplementary Note 1

Supplementary Discussions 1 to 6

Supplementary Figs. 1 to 12

Supplementary Tab. 1

Supplementary References

Other Supplementary Materials for this manuscript include the following:

Supplementary Movies 1 to 8

Supplementary Note 1. Characterization of surface wettability

The disc surface with a diameter d = 2 cm, as shown in the schematic diagram in Supplementary Fig. 1, consists of an array of concentric microgrooves with uniform ridge width W = 40 μm, groove width S = 40 μm and height H = 20 μm. As shown in Supplementary Fig. 2, a water drop of radius R = 1.18 mm was released gently on the microgrooved arrays at r = 5 mm at room temperature, and when the drop touches the substrate, it spreads rapidly along the curved hydrophilic grooves and reaches a stable wetting state at ~ 564 ms. After that, we measured the contact angle of several typical locations. Here, we denote θ_1 and θ_2 as the contact angles on the right and left sides of the cross-section of the arc-shaped drop along the radial direction. As show in the upper panel of Supplementary Fig. 2, we obtain $\theta_1 \approx 89.2° \pm 0.3°$, a large contact angle resulting from the contact line pinning at the corner of ridges[1]. However, we are not able to get a direct measurement of θ_2 as the left side of the cross-section is optically blocked. Considering the radius of the drop from the top view is quite large and the drop is not severely curved, $\theta_2 \approx \theta_1$ is expected. The third one is the contact angle θ_3 at the end of the drop, which is measured to be $\theta_3 \approx$ 32.5 ± 0.6°.

Supplementary Discussion 1. Drop deformation in the transition boiling state

As shown in Fig. 1b in the manuscript, shortly after the drop touches the substrate, it elongates and evolves into a bean shape (i.e., 9.8 ms). For simplicity, a model is built on the assumption that the cross-section of the bean shape is circular with a diameter e (see the right panel of Supplementary Fig. 4a).

Supplementary Discussion 2. Force analysis of the drop in the transition boiling state

From the top view in the left column of Supplementary Movie 1, the impact drop gradually

deviates from a circle during the spreading stage (i.e., 3.6 ms), and then changes into a bean shape at the recoiling state (i.e., 9.8 ms). The asymmetry of the left and right profiles of the droplet results in a Laplace pressure difference δP during the whole impact process. Consequently, the Laplace pressure difference forces a circular shape drop to become a bean-shape one. For simplicity, considering a bean-shape drop, a Laplace pressure difference $\delta P = \Delta P_{BA} = P_B - P_A$ is generated between the inner and outer liquid-vapour surfaces (Supplementary Fig. 5), which could be expressed as

$$P_B - P_0 \approx \gamma \left(\frac{1}{r_2} + \frac{1}{e/2} \right), \tag{S1}$$

$$P_A - P_0 \approx \gamma \left(-\frac{1}{r_1} + \frac{1}{e/2} \right), \tag{S2}$$

$$\Delta P_{BA} = P_B - P_A \approx \gamma \left(\frac{1}{r_1} + \frac{1}{r_2} \right), \tag{S3}$$

where P_0 is the pressure of the ambient atmosphere, and P_A and P_B are the pressure close to $r_1 = r - e/2$ and $r_2 = r + e/2$ in the bean-shape drop, respectively. Considering $e << r$, Eq. (S3) could be further written into

$$\delta P = \Delta P_{BA} \approx \frac{2\gamma r}{r^2 - (e/2)^2} \approx \frac{2\gamma}{r} \sim \frac{\gamma}{r}. \tag{S4}$$

Considering δP works on the drop along the horizontal direction during the whole recoiling stage, we obtain the force

$$F_L = \delta P \cdot A \sim \delta P \cdot D_{max}^2 \sim \frac{\gamma}{r} \cdot R^2 \mathrm{We}^{1/2}. \tag{2}$$

Supplementary Discussion 3. State of the drop in the film boiling state

At a higher temperature, i.e., film boiling state at $T = 350$ °C, the drop will rebound far away from the center of curvature (i.e., the right direction), as shown in Fig. 1c. In the following, we will devote to exploring the origin of the driving force.

Firstly, we estimate how depth the liquid could penetrate the grooves. Based on the geometrical relationships as shown in Supplementary Fig. 6, we have $R^* \sim S^2/\varepsilon$, where R^* and ε are the curvature radius and the penetration depth of the liquid-vapour meniscus, respectively. Thus, the Laplace pressure is $\Delta P_L \sim \gamma/R^* \sim \gamma\varepsilon/S^2$, which is balanced by the dynamics pressure $\rho U^2/2$ during the impingement. In this regard, we obtain

$$\varepsilon \sim \frac{S^2}{R}\mathrm{We}. \quad \text{(S5)}$$

Putting $S = 40$ μm, $R = 1$ mm and We = 10 into Eq. (S5), we obtain $\varepsilon \approx 16$ μm, which clearly show that the drop penetrates the grooves.

Supplementary Discussion 4. Force analysis of the drop in the film boiling state

Next, we deduce the underlying mechanisms accounting for the driving force. The drop recoils after reaching its maximum spreading. An acting force to the liquid would rise during the drop recoiling process, due to the penetration of the liquid into the grooves as suggested in Supplementary Discussion 3. For convenience, as shown in Supplementary Fig. 7a, we divide the drop into the left and right parts, marked with light red and blue colors, respectively. Here, we use red and blue arrows to represent the resistance resulting from the left and right grooves, respectively. At the bottom of the recoiling drop, all the grooves resist the motion of the liquid. Here, we employ μ to denote the resistive force per length (the unit is N/m).

Based on the above facts, we now calculate the resistive force offered by the bottom of the

recoiling drop. As shown in Supplementary Fig. 7b, for a specific groove with curvature radius $\bar{r}$, the resultant force along the X-direction can be obtained

$$F_{\bar{r}} = 2\mu\int_{S_0}^{S_1}\cos\psi \mathrm{d}S = 2\mu\bar{r}\int_0^{\theta}\cos\psi \mathrm{d}\psi = 2\mu\bar{r}\sqrt{1-\frac{\left(\bar{r}^2+r^2-R^2\right)^2}{4r^2\bar{r}^2}}, \tag{S6}$$

where $\theta = \arccos\left[\left(\bar{r}^2+r^2-R^2\right)/\left(2r\bar{r}\right)\right]$.

Therefore, the entire resultant force on the left part (light red color in Supplementary Fig. 7a) of the drop can be obtained as

$$\begin{aligned} F_{\text{left}} &= \frac{2\mu}{W+S}\int_{r-R}^{\sqrt{r^2-R^2}}\left(\int_{S_0}^{S_1}\cos\psi \mathrm{d}S\right)\mathrm{d}\bar{r} \\ &= \frac{2\mu}{W+S}\int_{r-R}^{\sqrt{r^2-R^2}}\bar{r}\cdot\sqrt{1-\frac{\left(r^2+\bar{r}^2-R^2\right)^2}{4r^2\bar{r}^2}}\,\mathrm{d}\bar{r} \end{aligned}. \tag{S7}$$

The above Eq. (S7) is too complicated and cannot be solved analytically. By employing Mathematica and taking considering of $R << r$, we find an approximate solution

$$F_{\text{left}} \approx \frac{2\mu}{W+S}\int_{r-R}^{\sqrt{r^2-R^2}}\frac{R}{r}\bar{r}\,\mathrm{d}\bar{r} \approx \frac{2\mu}{W+S}\left(R^2-\frac{R^3}{r}\right). \tag{S8}$$

Similarly, we can obtain the resistive force resulting from the right part of the drop (light blue color in Supplementary Fig. 7a) as

$$\begin{aligned} F_{\text{right}} &= \frac{2\mu}{W+S}\int_{\sqrt{r^2-R^2}}^{r+R}\left(\int_{S_0}^{S_1}\cos\psi \mathrm{d}S\right)\mathrm{d}\bar{r} \\ &\approx \frac{2\mu}{W+S}\int_{\sqrt{r^2-R^2}}^{r+R}\frac{R}{r}\bar{r}\,\mathrm{d}\bar{r} \\ &\approx \frac{2\mu}{W+S}\left(R^2+\frac{R^3}{r}\right) \end{aligned}. \tag{S9}$$

A combination of F_{right} and F_{left} leads to a net force

$$F_{\mathrm{R}} = F_{\mathrm{right}} - F_{\mathrm{left}} \approx \frac{2\mu}{W+S} \cdot \frac{2R^3}{r} \sim \frac{\mu R^3}{(W+S)r} . \tag{S10}$$

Equation (S10) clearly shows that F_{R} is pointing to the right, that the drop will rebound far away from the center of the curvature, which fully agrees with our experimental observation.

The stop of the flow blocked by the inner wall of the groove provides the resistive force, so $\mu \sim \rho U_{//}^2 H$. As shown in Supplementary Fig. 8, $U_{//}$ represents the recoiling speed of the drop, which obeys the Taylor-Culick velocity, i.e., $U_{//} \sim [\gamma/(\rho h)]^{1/2}$, with $h = R\mathrm{We}^{-1/2}$ being the characteristic thickness of the drop at its maximum spreading diameter $D_{\mathrm{max}} \sim D\mathrm{We}^{1/4}$. Such, we obtain $\mu \sim \gamma H \mathrm{We}^{1/2}/R$ and

$$F_{\mathrm{R}} \sim \frac{R^2 H \gamma}{(W+S)} \cdot \frac{\mathrm{We}^{1/2}}{r} . \tag{4}$$

Supplementary Discussion 5. Drop lateral displacement at high temperatures

Based on the net force F_{net} exerted on the drop along the lateral direction, we can estimate the lateral displacement Δl as follows

$$\Delta l \sim \frac{F_{\mathrm{net}}}{m} \cdot \tau_0 \cdot \frac{U_{\perp}}{g} . \tag{S11}$$

In the above Eq. (S11), $m = 4\pi\rho R^3/3$ is the mass of the drop and $\tau_0 \sim (\rho R^3/\gamma)^{1/2}$ is the solid-liquid contact time, and therefore, $(F_{\mathrm{net}}/m)\tau_0$ represents the characteristic speed of the drop along the lateral direction. $U_{\perp} = [\gamma/(\rho R)]^{1/2}$ and $U_{\perp}/g$ characterize the rebounding velocity and the duration of the free-fall drop, respectively.

When $T = 250$ °C, changing F_{net} into F_{left} and substituting Eq. (2) into Eq. (S11), we obtain

$$\Delta l_{\mathrm{L}} \sim l_{\mathrm{c}}^{2} \frac{\mathrm{We}^{1/2}}{r} . \quad (3)$$

When $T = 350$ °C, chaning F_{net} into F_{right} and substituting Eq. (4) into Eq. (S11), we obtain

$$\Delta l_{\mathrm{R}} \sim \frac{H l_{\mathrm{c}}^{2}}{W + S} \cdot \frac{\mathrm{We}^{1/2}}{r} . \quad (5)$$

Supplementary Discussion 6. Effect of structural parameters on the drop lateral transport

Substrates with different parameters are shown in Supplementary Fig. 9. Since there are no generally valid parameter settings in parameter selection, the parameters were selected based on the preliminary investigations. In the drop impact experiments on these substrates, the off-center distance between the impact point and center of curvature is fixed at $r = 5$ mm.

In the following, we will further elaborate the effect of structural parameters on drop lateral transport. At $T = 250$ °C, the impacting drop on all the substrates is in the transition boiling state. As shown in Supplementary Fig. 10a, for a given We, the maximum spreading diameter of drops D_{max} impacting on substrates with different structure parameters remains constant, resulting in the scaling law $D_{\mathrm{max}}/D \sim \mathrm{We}^{1/4}$ with a prefactor ranging between 1.25 and 1.34. Following the discussion in the manuscript, the driving force that propels the drop to the curvature center of the substrate could be deduced based on the asymmetric profiles of the drop, and the lateral distance of impacting drop could be scaled as $\Delta l_{\mathrm{L}} \sim l_{\mathrm{c}}^{2} r^{-1} \mathrm{We}^{1/2}$, where l_{c}, We and r are the capillary length, Weber number and the off-center distance between the impact position and center of curvature, respectively. The experimental result in Supplementary Fig. 10b shows a good agreement with the derived model, and the prefactor for the best fit is 0.35. Note that, the structural parameters have little influence on the lateral displacement of drop.

At T = 350 °C, the impacting drop on all the substrates with different parameters is in the film boiling state. As shown in Supplementary Fig. 10c, it is obvious that Δl_R increases with We, which is in a good agreement with the derived model of Eq. (5) in the manuscript, except the data points on substrates W20S10H20, W40S10H20 and W40S40H50. We will explain the reasons as follows. Based on the estimation of Eq. (S5), on substrates W20S10H20 and W40S10H20 with S = 10 μm, the penetration depth of the liquid-vapour meniscus is estimated to be $\varepsilon \sim (S^2/R)$We. For example, $\varepsilon \approx 1$ μm is obtained for R = 1 mm and We = 10, which suggests that the amount of liquid penetrated into the groove is too small to generate sufficient lateral interaction force between the drop and the microgrooved substrate and therefore an unobvious lateral displacement is observed, as shown in Supplementary Fig. 10c.

On the contrary, a befittingly large groove height, e.g., H = 50 μm, retains a suitable space making vapour rectification non-negligible[2]. However, the direction of the lateral force resulting from the vapour flow is opposite to the direction of the force resulting from the interaction between the drop and the microgrooves. In this regard, the net lateral force that the drop experiences on the substrate W40S40H50 is a combination of two forces: (i) a driving force towards the direction far away from the center of curvature, which is generated between the grooves and the penetrated liquid; (ii) a shear force resulting from the viscosity of gas towards the center of curvature, which is generated from the vapour flow between the drop and the hot substrate. The viscous stress τ_{vis} between the drop and the hot substrate is

$$\tau_{vis} \sim \eta \frac{U_a}{\delta}, \tag{S12}$$

in which η is the viscosity of gas, U_a is the gas velocity between the drop bottom and the substrate, and δ is the gas thickness and here it is characterized by the depth of the groove. Based on thermodynamics[2], we obtain

$$U_a = \frac{k \cdot \Delta T}{\rho_a L \delta^2} \cdot x, \tag{S13}$$

in which ρ_a is the vapour mass density, ΔT is the temperature difference between the substrate and the liquid boiling point, k is the thermal conductivity of the vapour, L is the latent heat of evaporation and x is the position of the channel, i.e., $x \approx r_i\varphi$ with $0 < \varphi < \varphi_i$, as shown in Supplementary Fig. 11.

Next, we will consider the viscous force resulted from each channel that the drop occupies at the contact region, and then calculate the entire shear force. In the region A_i (enclosed by the red line) with a distance r_i far from the substrate center as shown in Supplementary Fig. 11b, the lateral shear force F_i (pointing to the substrate center) exerted on the liquid-gas interface of the drop is

$$F_i = \tau_{vis} \left(A_i f_a \right) \sin \varphi_i, \tag{S14}$$

with

$$f_a + f = 1, \tag{S15}$$

where f_a and f are defined as the area fractions of the liquid-gas area and the solid-liquid area on the contact region, respectively. In our experiments, $f_a = S/(W + S)$. After that, the entire shear force could be obtained by making an integral over the whole contact region

$$\begin{aligned} F_{vis} &= \sum_{i=1}^{n} \tau_{vis} \left(A_i f_a \right) \sin \varphi_i \\ &= \sum_{i=1}^{n} \frac{\eta}{\delta} \left(\frac{k \cdot \Delta T}{\rho_a L \delta^2} \cdot x \right) A_i f_a \sin \varphi_i \end{aligned}. \tag{S16}$$

For the sake of simplicity, here we employ a scaling analysis to handle Eq. (S16). Specifically, the following simplifications are considered

$$x \sim \frac{R_{\max}}{2}, \quad \sum_{i=1}^{n} A_i \sim R_{\max}^2, \quad \sin\varphi_i \sim \frac{R_{\max}}{r}, \tag{S17}$$

and then we obtain

$$F_{\text{vis}} = \frac{\eta \cdot k \cdot \Delta T}{\rho_a L \delta^3} \cdot \frac{R_{\max}^4}{r} \cdot f_a. \tag{S18}$$

Therefore, the force F_{net} acting on the impinging drop could be expressed as a combination of F_{vis} and $F_R \sim R^2 \gamma H (W+S)^{-1} \mathrm{We}^{1/2} r^{-1}$, e.g., $F_{net} = a_1 F_R - a_2 F_{vis}$ with a_1 and a_2 being numerical coefficients,

$$F_{\text{net}} = a_1 \cdot \frac{R^2 H \gamma}{(W+S)} \cdot \frac{\mathrm{We}^{1/2}}{r} - a_2 \cdot \frac{\eta \cdot k \cdot \Delta T}{\rho_a L \delta^3} \cdot \frac{R_{\max}^4}{r} \cdot f_a. \tag{S19}$$

Putting Eq. (S19) into Eq. (S11), we could obtain the modified lateral displacement $\Delta l_R'$

$$\begin{aligned} \Delta l_R' &= \frac{F_{\text{net}}}{m} \cdot \tau_0 \cdot \frac{U_\perp}{g} \\ &= \left(a_1 \cdot \frac{R^2 H \gamma}{(W+S)} \cdot \frac{\mathrm{We}^{1/2}}{r} - a_2 \cdot \frac{\eta \cdot k \cdot \Delta T}{\rho_a L \delta^3} \cdot \frac{R_{\max}^4}{r} \cdot f_a \right) \cdot \frac{3}{4\rho\pi R^3} \cdot \sqrt{\frac{\rho R^3}{\gamma}} \cdot \frac{1}{g} \cdot \sqrt{\frac{\gamma}{\rho R}}. \\ &= a_1 \cdot \frac{H l_c^2}{W+S} \cdot \frac{\mathrm{We}^{1/2}}{r} - a_2 \cdot \frac{\eta \cdot k \cdot \Delta T}{\rho_a L \delta^3} \cdot \frac{R^2 l_c^2}{\gamma} \cdot f_a \cdot \frac{\mathrm{We}}{r} \end{aligned} \tag{S20}$$

As shown in Supplementary Fig. 10d, by including the effect of the gas flow under the drop during impingement, the modified lateral distance $\Delta l_R'$ estimated by Eq. (S20) is very well consistent with the experimental data extracted from the substrate W40S40H50, with a_1 = 0.21 and a_2 = 0.14 based on the best fit.

Supplementary Figures

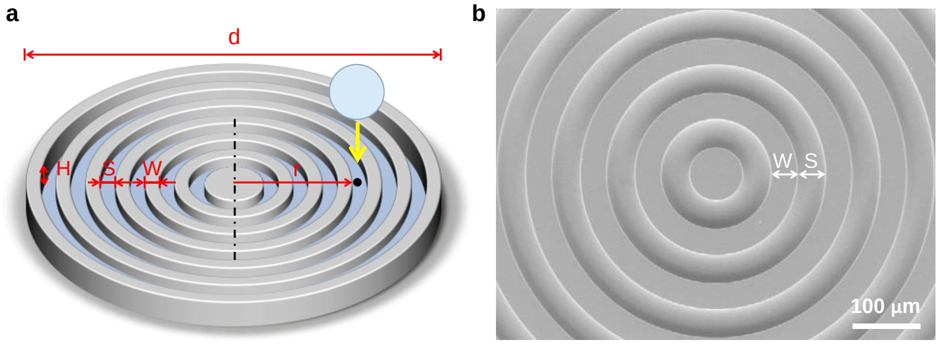

Supplementary Fig. 1 Substrate with concentric microgroove arrays. a Schematic showing a drop impacting on the substrate consisting of concentric microgroove arrays. The width, height and spacing of the ridge are $W = 40$ μm, $S = 40$ μm and $H = 20$ μm, respectively, and labelled as W40S40H20. r is the distance between the impact point and the center of curvature, and d is the diameter of the substrate. **b** Scanning electron microscope image of the surface from the top view.

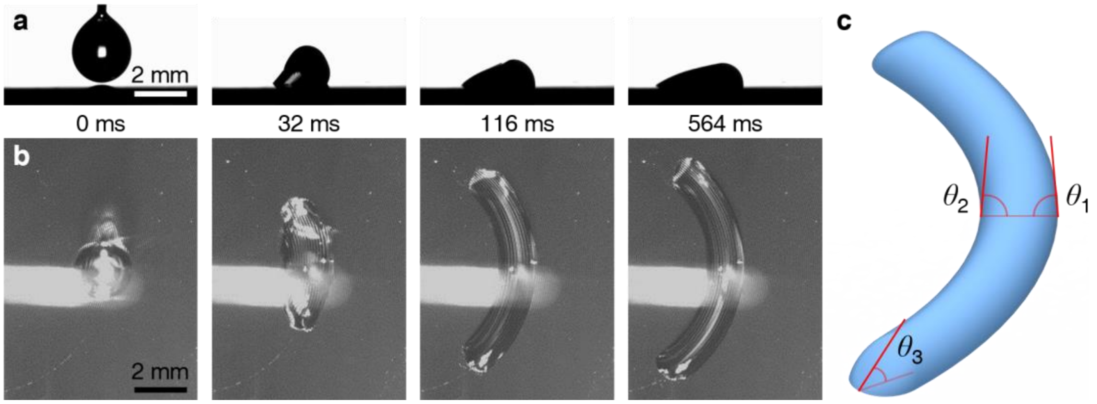

Supplementary Fig. 2 Characterization of wettability. a, b Selected snapshots showing the anisotropic spreading of a drop gently deposited on the microgroove arrays at *r* = 5 mm at room temperature in both the side (**a**) and top (**b**) views. **c** Schematic showing the contact angles at three typical locations.

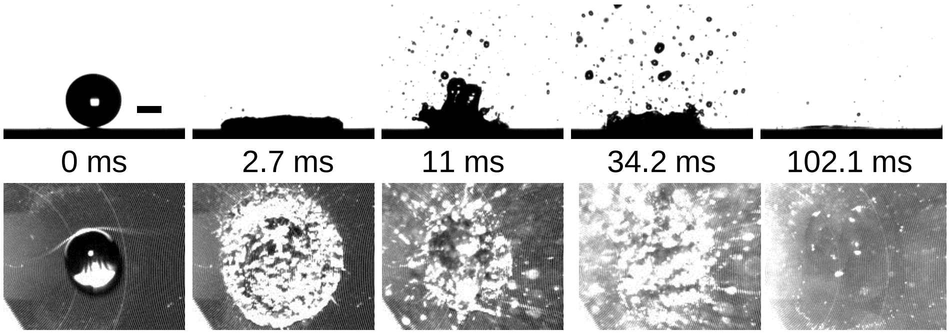

Supplementary Fig. 3 Drop dynamics in the contact boiling state. Side (upper panel) and top (lower panel) views showing the water drop impact process on concentric microgroove arrays, where We = 16.9, $r = d/4$ and $T = 200$ °C. Scale bar is 1 mm.

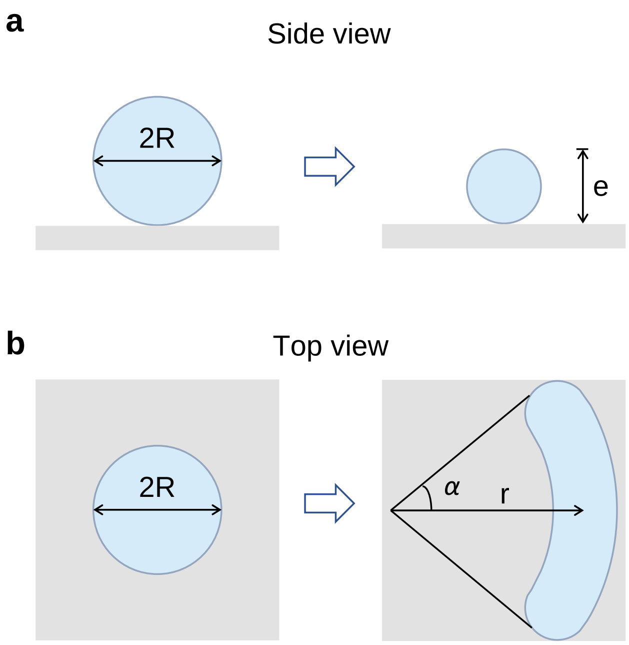

Supplementary Fig. 4 Morphology of recoiling drop on concentric microgroove arrays at the transition boiling state. a, **b** Schematics showing the drop shape at the moments that the drop just touches the substrate (left panel) and reaches a bean shape (right panel) from the side view (**a**) and top view (**b**), respectively. Relevant geometrical parameters R, r, α and e, are defined.

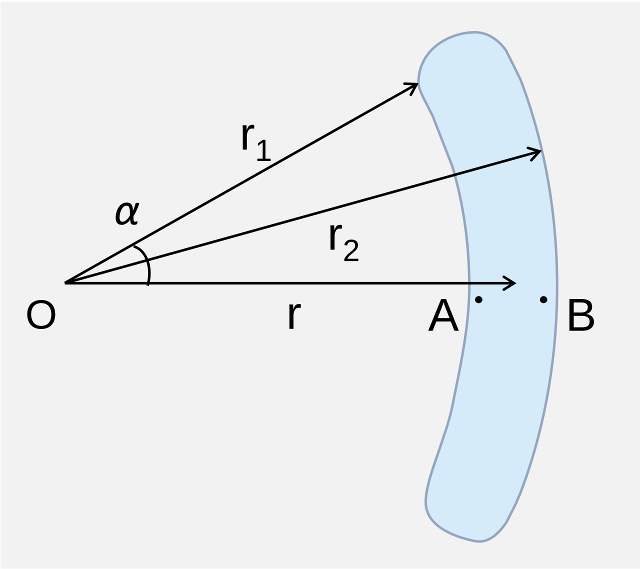

Supplementary Fig. 5 Schematic showing the origin of the driving force. Relevant geometrical parameters are defined. Points A and B denote the representative points close to the left and right menisci of the drop.

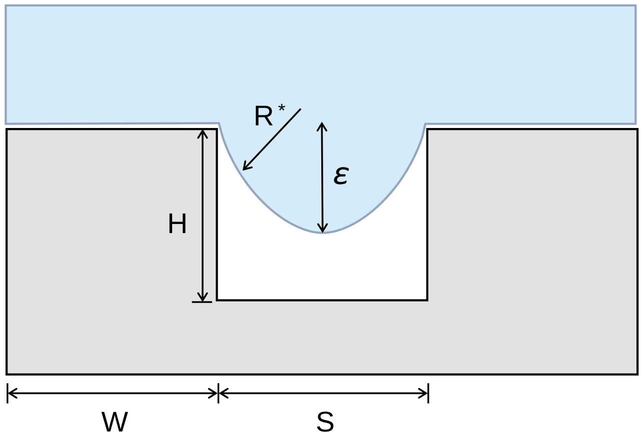

Supplementary Fig. 6 Schematic depicting the deformation of the liquid-vapour meniscus during the impingement. Relevant geometrical parameters are marked.

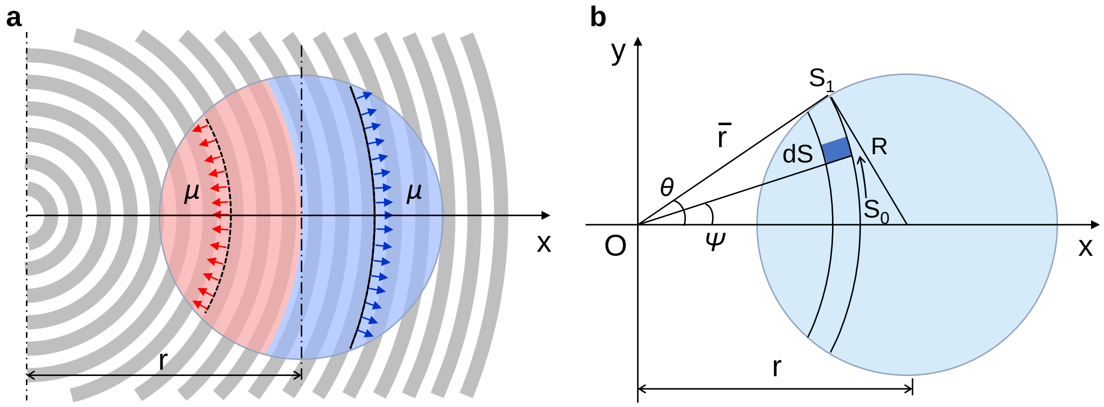

Supplementary Fig. 7 Schematics showing the geometry and modelling of the recoiling drop.
a The recoiling drop is divided into two parts and marked with light red (the left part) and yellow (the right part) colors, respectively. The red (left part) and blue (right part) arrows represent the resistive forces from the inner walls of the grooves. **b** Theoretical model for calculating the entire resistive force offered by the bottom of the recoiling drop.

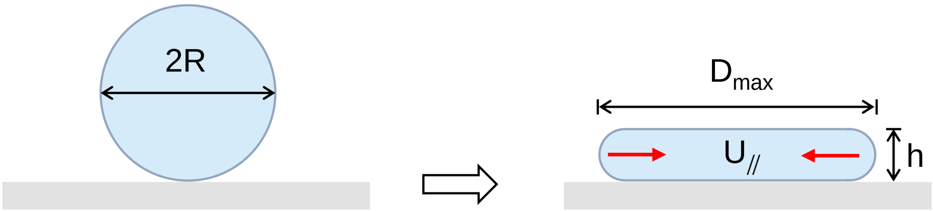

Supplementary Fig. 8 Schematic showing the shape transition of the drop from the side view. Left: the moment that the drop just touches the substrate. Right: the moment that the drop reaches its maximum spreading diameter D_{max}. The drop recoils with a speed $U_{//}$ and a thickness h.

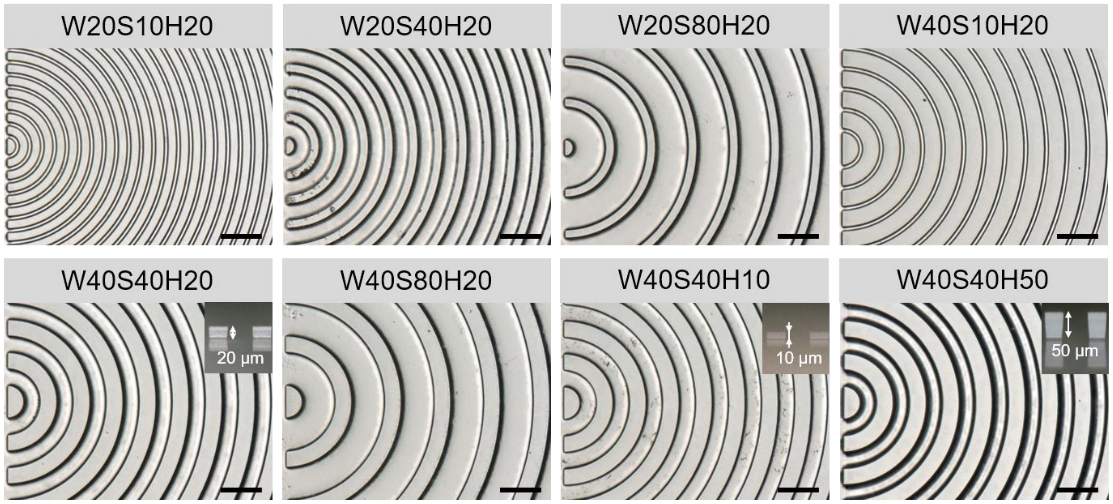

Supplementary Fig. 9 Optical images of substrates with different structure parameters. All scale bars represent 100 μm.

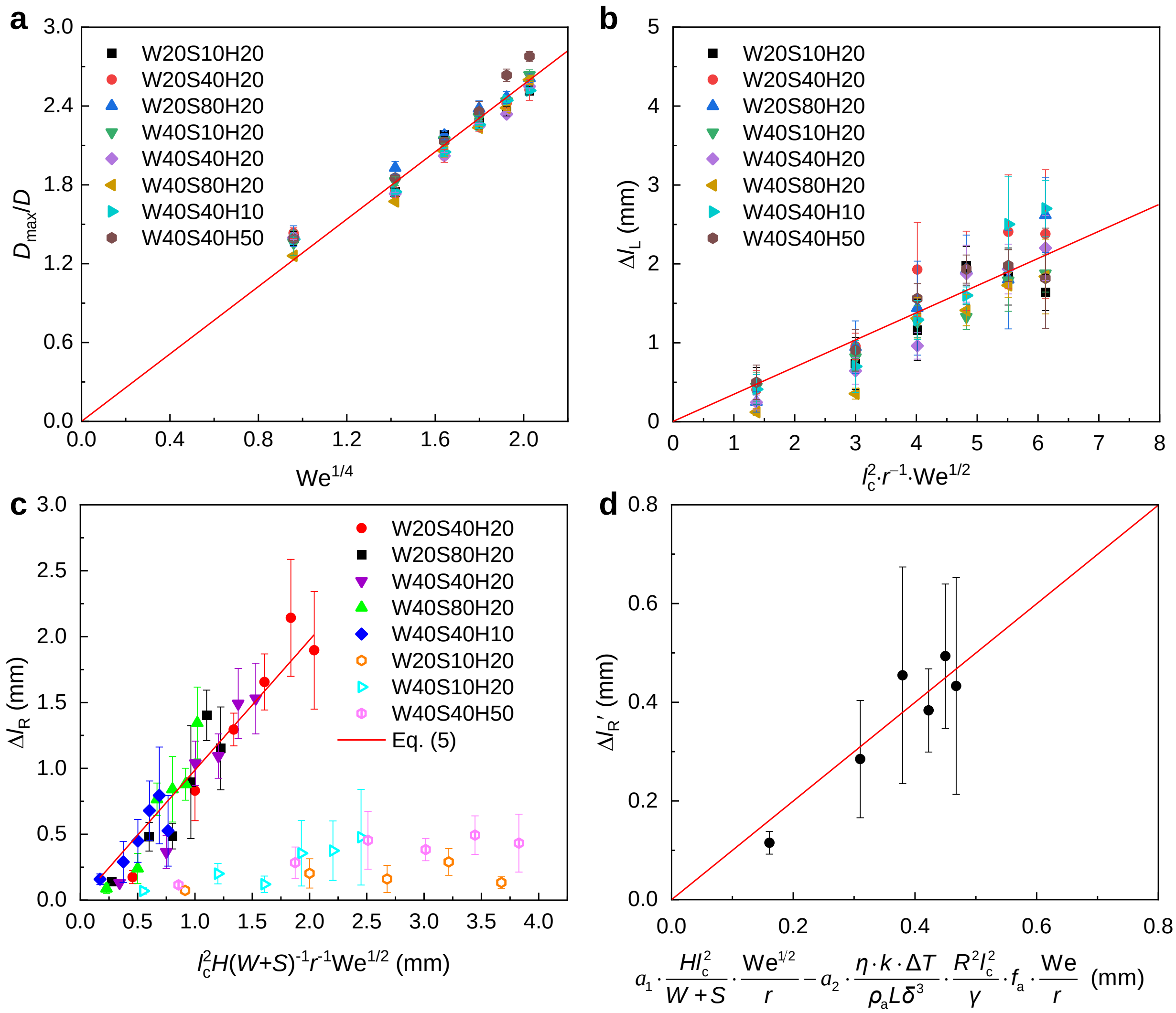

Supplementary Fig. 10 Drop lateral transport on substrates with different structure parameters. a Relationship between D_{max}/D and $We^{1/4}$ on substrates with different structure parameters. The red line denotes the best fit to all the experimental data. The error bars denote the standard deviation of the measurements. **b** Lateral displacement Δl_L as a function of structural parameters at T = 250 °C. **c** Lateral displacement Δl_R as a function of structure and collision parameters at T = 350 °C. The error bars denote the standard deviations of five measurements. **d** Comparison between the experimental and theoretical results of the lateral displacement of an impacting drop on the surface W40S40H50. The error bars of data in (**a**)-(**d**) denote the standard deviations of five measurements. Source data are provided as a Source Data file.

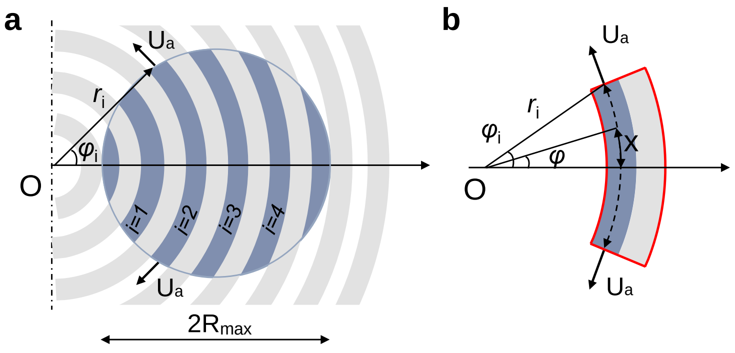

Supplementary Fig. 11 Schematics showing the geometry and modelling of the vapour flow between the drop and the hot substrate. The light grey color represents the ridge, while the dark blue color represents the groove under the impinging drop. **a** The vapour flow escapes along the curve grooves. **b** Theoretical model for calculating the entire shear force offered by the vapour flow.

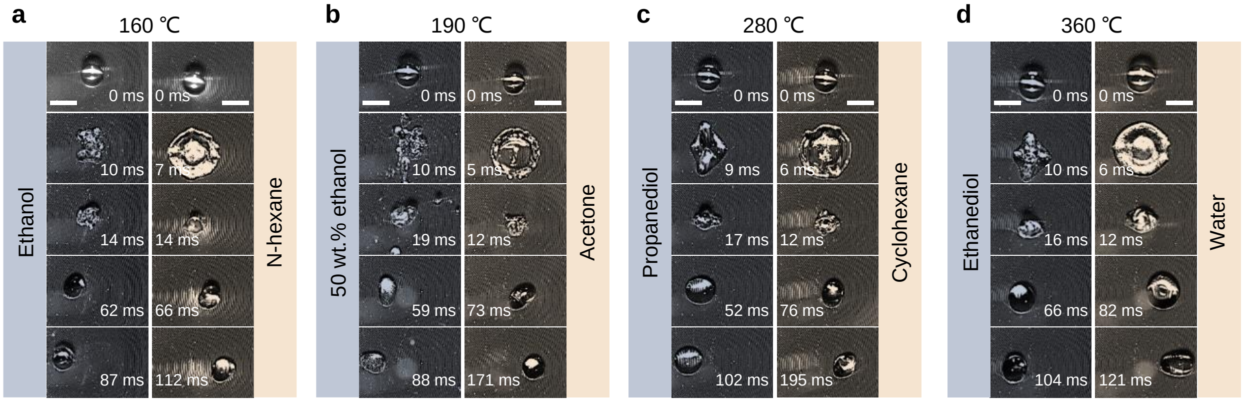

Supplementary Fig. 12 Sequential images showing opposite rebounding directions of drops at a certain temperature. a The ethanol drop bounces towards the center of curvature while the n-hexane drop bounces far away from the center of curvature at 160 °C. **b** The 50 wt.% ethanol drop bounces towards the center of curvature while the acetone drop bounces far away from the center of curvature at 190 °C. **c** The propanediol drop bounces towards the center of curvature while the cyclohexane drop bounces far away from the center of curvature at 280 °C. **d** The ethanediol drop bounces towards the center of curvature and the water drop bounces far away from the center of curvature at 360 °C. All scale bars are 2 mm.

Supplementary Table

Supplementary Tab. 1 Physical properties of various liquids at 20 °C

Properties	ethanediol	propanediol	water	50 wt.% ethanol	ethanol	cyclohexane	acetone	N-hexane
Surface tension ($mN\cdot m^{-1}$)	48.4	36	72.8	29.5	22.32	25.3	18.8	17.89
Density ($g\cdot cm^{-3}$)	1.113	1.0381	0.998	0.91	0.789	0.791	0.7899	0.66
Viscosity (mPa·s)	21.38	56	1.01	2.87	1.17	0.94	0.32	0.31
Boiling point (°C)	197.3	184.8	100	81.9	78.3	80.72	56.5	69

Supplementary References

1. Y. V. Kalinin, V. Berejnov, R. E. Thorne, Contact line pinning by microfabricated patterns: Effects of microscale topography. *Langmuir* **25**, 5391–5397 (2009).
2. D. Soto, G. Lagubeau, C. Clanet, D. Quéré, Surfing on a herringbone. *Phys. Rev. Fluids* **1**, 013902 (2016).
