# Supplementary material for: Steerable drops on heated concentric microgroove arrays
Source: Nat Commun. 2022 Jun 6;13:3141. doi: 10.1038/s41467-022-30837-z (PMC9170727; doi:10.1038/s41467-022-30837-z)
Supplement: Supplementary file 2 — Description of Additional Supplementary Files [file 41467_2022_30837_MOESM2_ESM.pdf]

Description of Additional Supplementary Files

File Name: Supplementary Movie 1
Description: The side and top views of a drop impinging on concentric microgroove arrays. The impinging water drop bounces towards the center of curvature at T = 250 °C, We = 16.9 and $r = d/4$ (Left column). The impinging drop bounces towards the direction far away from the center of curvature at T = 350 °C, We = 16.9 and $r = d/4$ (Right column).

File Name: Supplementary Movie 2
Description: The convergence of randomly released water drops. The randomly released water drops converge into the center of curvature of the concentric microgroove arrays at T = 250 °C and We = 7.3.

File Name: Supplementary Movie 3
Description: The convergence of successive impinging drops with a fixed releasing point. Successive water drops converge into the center of curvature of the concentric microgroove arrays at T = 250 °C and We = 10.5.

File Name: Supplementary Movie 4
Description: Opposite rebounding of impinging ethanol and n-hexane drops. The impinging ethanol drop bounces towards the center of curvature (Left column), while the impinging n-hexane drop bounces towards the opposite direction (Right column). Here, T = 160 °C, We = 10.5 and $r = d/4$.

File Name: Supplementary Movie 5
Description: Drop sieving. The synchronously deposited drops (ethanol and n-hexane) rebound to opposite directions showing the ability of drop sieving at We = 10.5, T = 160 °C and $r = d/4$. The ethanol drop bounces towards the center of curvature, while the n-hexane drop bounces towards the opposite direction.

File Name: Supplementary Movie 6
Description: Opposite rebounding of impinging aqueous ethanol and acetone drops. The impinging 50 wt.% ethanol drop bounces towards the center of curvature (Left column), while the impinging acetone drop bounces towards the opposite direction (Right column). Here, T = 190 °C, We = 10.5 and $r = d/4$.

File Name: Supplementary Movie 7
Description: Opposite rebounding of impinging propanediol and cyclohexane drops. The impinging propanediol drop bounces towards the of curvature (Left column), while the impinging cyclohexane drop bounces towards the opposite direction (Right column). Here, T = 280 °C, We = 10.5 and $r = d/4$.

File Name: Supplementary Movie 8
Description: Opposite rebounding of impinging ethanediol and water drops. The impinging ethanediol drop bounces towards the center of curvature (Left column), while the impinging water drop bounces towards the opposite direction (Right column). Here, T = 360 °C, We = 10.5 and $r = d/4$.
